# Online Knowledge-Based Model for Big Data Topic Extraction

**DOI:** 10.1155/2016/6081804

**Published:** 2016-04-19

**Authors:** Muhammad Taimoor Khan, Mehr Durrani, Shehzad Khalid, Furqan Aziz

**Affiliations:** ^1^Bahria University, Shangrilla Road, Sector E-8, Islamabad 44000, Pakistan; ^2^FAST-NUCES, Industrial Estate Road, Hayatabad, Peshawar 25000, Pakistan; ^3^COMSATS IIT, Kamra Road, Attock 43600, Pakistan; ^4^IMSciences, Phase 7, Hayatabad, Peshawar 25000, Pakistan

## Abstract

Lifelong machine learning (LML) models learn with experience maintaining a knowledge-base, without user intervention. Unlike traditional single-domain models they can easily scale up to explore big data. The existing LML models have high data dependency, consume more resources, and do not support streaming data. This paper proposes online LML model (OAMC) to support streaming data with reduced data dependency. With engineering the knowledge-base and introducing new knowledge features the learning pattern of the model is improved for data arriving in pieces. OAMC improves accuracy as topic coherence by 7% for streaming data while reducing the processing cost to half.

## 1. Introduction

Machine learning (ML) topic models are popularly used for different Natural Language Processing (NLP) related tasks. Although it ignores word semantics, topic models provide better results with least user involvement. Unsupervised topic models are frequently used for domain exploration in many research areas where training data is not available or is expensive to produce. Topic models are the statistics based techniques, evaluating words cooccurrence probabilities, where the results improve with the size of data. The relation based techniques struggle with the richness of natural language, to explore all possible forms that a word can have. Lifelong learning models put an end to the traditional single-shot learning with the aim of improving the accuracy of single-domain data. The big textual data has large volume and has variety of domains discussed and the data is being poured in continuously and is expected to have inconsistencies and noise. Keeping up with the amount and flow of content, lifelong learning models can help build practical applications.

The unsupervised topic models extract incoherent topics as well along with the coherent topics [1–5]. Different extensions of topic models are proposed to improve their accuracy. The semisupervised topic models used manually provided seed aspects to help find more relevant product aspects [6–9]. Supervised topic models are trained for a domain to achieve high accuracy at it. Hybrid models are trained on small labeled data to predict suitable initial values for topic model [10–15]. Knowledge-based topic models are guided by domain experts with knowledge rules instead of seed terms to deal with incoherent topics [16–18]. Transfer-learning based topic models are trained on one domain and tested on another single target domain closely related to it that is known prior to analysis. However, all of these models dealt with improving the accuracy for a single domain. Therefore, they lack scalability and cannot be used for big data consisting of multiple unknown domains. Despite their high accuracy, these models have limited application as they are devised for a single-domain analysis.

Lifelong machine learning model takes a completely new dimension to use a model that can process many unknown domains. It incorporates automatically generated knowledge to improve the quality and coherence of topics without requiring any external support. The model maintains its own knowledge-base that grows with experience and so the model matures in decisions to attain improved accuracy. LML models are the computational systems that process tasks and retain popular patterns from it. This approach is also called human-like learning or never ending learning in the literature. Lifelong learning is only recently introduced to NLP tasks. Never Ending Language Learner (NELL) is considered the first attempt to use lifelong learning for NLP [19]. It extracts information from web sources to extend a knowledge-base in an endless manner, aiming to achieve improved performance with experience. Unlike other machine learning approaches, there is little research work available on lifelong machine learning for NLP related tasks.

There are four main components of LML models that are knowledge representation, extraction, transfer, retention, and maintenance, discussed in [20]. These components are strongly connected and analyzing them for quality improvement is called knowledge engineering. The knowledge learnt is represented through an abstracted set of features. The extraction component focuses on identifying popular patterns and mining them as potential knowledge. The transfer component is responsible for transferring the impact of knowledge into the modeling process when suitable. The retention and maintenance module deals with storing knowledge for future use. The objective is to use knowledge to reduce the number of incoherent topics. Topic coherence means that the words in a topic do not semantically hold well together. Topic incoherence can be of various types, that is, chained, intruded, random, and unbalanced, as explained by [21]. Chained problem refers to the case where there are word pairs having strong interconnection forming a chain but there is no collective relatedness among the words of a topic. Intruded topics have set of words with good corelation within the set but not fitting well with the rest of the words in the topic. Random topics do not make any sense while unbalanced topics have a mix of general and specific terms that does not hold good together. With the incoherence problems known, the modules of LML model can be modified to closely focus on them.

Lifelong learning topic models are introduced to benefit from the knowledge-base while keeping it independent of domain specific manual tuning. This makes it possible for topic models to mine topics from big data with many domains. Automatic Knowledge Learning (AKL) model [22] is the first such model that used automatic knowledge learning for topic models. Later, there are different lifelong learning models proposed where the process of knowledge extraction and transfer is refined for improved accuracy and performance. However, they still have limitations to be addressed. The existing LML models lack a knowledge maintenance and retention module. Therefore, they have to load topics from all tasks performed to sample relevant knowledge for the current task. The knowledge learnt is limited which was initially consisting of must-links as word pairs having strong positive corelation. In later models cannot-links were also added as word pairs with strong negative corelation. Following batch processing approach, they expect the big data to be available prior to analysis. If topics of an already performed task are removed, the learning curve of the model is affected. Therefore, existing state-of-the-art models consume more storage and processing resources and do not provide support for streaming data arriving in pieces.

The proposed model is online, that is, processing one domain at a time, as they are received. Each domain is processed only once, with the knowledge available at that time. After processing each domain, the knowledge-base is updated. With a knowledge retention module, it does not require loading all the topics from the previously processed domains. It makes the model lightweight without compromising accuracy. The model has low data dependency where it only has to load the occurrences of relevant knowledge maintained in abstract form. The contributions of this paper are highlighted below.

## 2. Highlights

(1) With online approach, it processes the data as it is received in intervals. It makes the proposed model closer to the essence of big data streaming from different sources.

(2) Knowledge retention module is introduced to store knowledge in abstract form. Unlike existing models to load topics from all domains, it only consults the knowledge-base, thus reducing dependency on data. Therefore, it is efficient in performance and resource utilization.

(3) Must-links and cannot-links are extracted with a single mechanism, that is, normalized Pointwise Mutual Information (PMI) as nPMI [23]. They are stored, maintained, and transferred irrespective of their orientation for improved performance.

(4) Being online the proposed model has limited data at a time to learn from and is expected to learn low quality knowledge. Knowledge freshness, utility, and confidence are introduced to monitor, prioritize, and even filter out knowledge if not contributing considerably.

(5) Knowledge representation and abstraction are improved to resolve word sense disambiguation without consulting all the processed domains. Knowledge rules are supported with background, representing top topic words from which the rule is mined. Reasonable overlap between the current domain and a knowledge background suggests same word sense.

## 3. Related Work

Probabilistic topic models based on Latent Dirichlet Allocation (LDA) extract corelations among words through statistical computations by calculating probabilities of words coexistence [24]. It uses Markov Chain Monte Carlo (MCMC) inference to combine words under topics having a thematic relevance. Following bag-of-words (BOW) approach it considers words presence and their positions. Initially introduced with variational Bayesian technique, mostly Gibbs sampling is used for inference to converge the analysis towards more probable groupings in the given number of iterations mostly kept high in thousands. The accuracy of topic models relies on the initial values for hyperparameters as well; however, it generally performs well for larger datasets. Probabilistic Latent Semantic Analysis (pLSA) [25] having extensions is also used for topic modeling; however, LDA is preferred for adjustable priors to closely refer to the nature of specific documents and topics. LDA holds the advantage of identifying and aggregating topics together, where they are separate steps in other approaches, for example, dictionary based and relation based. The extensions of unsupervised LDA used for aspect and sentiment terms extraction as topics are [1–5]. The hybrid topic models used for topic extraction and sentiment analysis are [10–15]. The semisupervised LDA models guided with initial seed aspect terms for topic extraction as product aspects are [6–9].

To improve the accuracy of topic models while keeping the user involvement to minimum, knowledge-based semisupervised models are proposed. The purpose of these models is to provide domain specific knowledge rules that guide the extraction process to reduce the number of incoherent topics extracted. Dirichlet Forest priors based LDA (DF-LDA) was introduced as a knowledge-based topic model, to which knowledge rules are provided in the form of must-link and cannot-link [16]. It changed the perspective towards topic extraction as the accuracy was dependent on the quality of knowledge. The knowledge rules guided the model to put terms under the same or different topics based on the intuition. A must-link increases the probability of word pairs to coexist under the same topic while a cannot-link works opposite to it. DF-LDA, being transitive, could not handle multiple senses of a word, which was not addressed in their later model [17] as well. The knowledge rules are assumed to be accurate, with no refinement mechanism, and are applied with equal priority. The rules were relaxed to insets in [18], having topic labels for words and thus avoiding one-to-one relationships for word coexistence. These semisupervised knowledge-based topic models are manually provided with domain specific knowledge and cannot scale up to big data. The knowledge provided is assumed to be accurate, having no mechanism for verifying and rectifying knowledge, if provided by mediocre domain experts.

Lifelong machine learning is used for various tasks in the works of [26–29]; however, none of them has used LML for topic modeling. Automatic knowledge-based models are introduced to incorporate a knowledge module with topic models to have more semantically corelated topics. The automatic LML models learn and apply knowledge automatically without external support and can be directly applied to big data. Overlap among different domains is exploited for learning and transferring knowledge. AKL [22] was the first attempt to introduce lifelong machine learning with topic modeling. The model processes all the domains in the dataset with baseline LDA to extract initial topics. The extracted topics are clustered to learn and utilize knowledge. Lifelong Topic Model (LTM) [30] improved the performance and accuracy of LML topic mining by using Frequent Itemset Mining (FIM) for knowledge extraction and Generalized Polya Urn (GPU) model for transferring it to future task. Manual support was provided to set threshold for Frequent Itemset Mining (FIM); however, it could not address the problem of mining local knowledge. A large threshold value means mining only global knowledge while a low value that focuses on local knowledge brings many irrelevant knowledge rules as well. They both used positively corelated words only as knowledge, that is, must-links.

In [20] automatic must-links cannot-links (AMC) model is proposed to address the problems of earlier automatic LML models. Unlike other LML models it used both must-link and cannot-link knowledge types. The knowledge rules are extracted with FIM with multiple support (MS-FIM) to consider both global and local knowledge while avoiding irrelevant knowledge rules. Since the cannot-links are believed to be very high due to the large size of vocabulary the model only extracts cannot-links for the current domain. In order to ensure their quality, the must-links and cannot-links are verified from topics of all domains. For transferring the learnt knowledge into the inference technique Multigeneralized Polya Urn (M-GPU) model is used. For a word drawn from a topic, if there are relevant must-links all the paired words get a pushup in the topic whereas a penalty is applied to the paired words in cannot-links. M-GPU provides the bias supported by knowledge in order to help distributions with topics having semantically corelated words. The transitivity problem is addressed with a graph connecting knowledge rules that share a common word which is used in the same sense.

The existing state-of-the-art LML models follow batch processing approach. They expect all the domains to be available prior to analysis. The big data is processed with baseline LDA to extract topics in first parse. In the second parse each domain is processed again with knowledge. Topics from all domains are loaded for each task to learn relevant knowledge that is verified from many domains. It makes these models highly dependent on data, as they require topics from all domains to be available at all times. All the knowledge rules extracted are treated equally, whereas some knowledge rules have high confidence while others marginally cross threshold. There is no mechanism to prioritize knowledge rules. These models lack a knowledge maintenance module and have to load topics from all domains for each task and thus consume more resources.

## 4. Proposed Model (OAMC)

**Figure 1:**
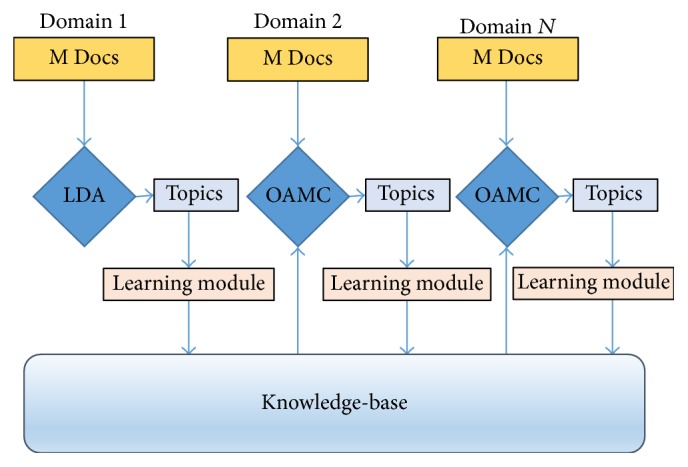
Proposed model (OAMC) using online knowledge extraction and transfer mechanism.

An online LML model (OAMC) is proposed to address the issues discussed. It is more closely related to the essence of big data analysis by expecting data to arrive continuously in chunks. It is less dependent on data, therefore consuming limited storage and processing resources. Efficiency of the model is associated with the knowledge-base instead of topics from all domains. With a newly introduced knowledge maintenance and retention module, the harvested knowledge rules are stored in an abstract form. The knowledge-base is maintained after each task to keep it consistent. New knowledge rules are mined while existing rules are updated or even filtered as a continuous process. Additional features of knowledge are introduced to help maintain and prioritize them. The working of the model is shown in Figure 1. With the online sources producing textual content continuously, the proposed model can keep up with processing needs of the data in hand. This makes it more practical for developing real-world monitoring and analysis applications.

The OAMC model can operate both with and without knowledge. In case there is no relevant knowledge available, the model operates as baseline LDA. However, as the model matures, its knowledge-base grows to support a variety of future tasks. With relevant knowledge, the model shows improved performance by grouping semantically related words under topics with the help of knowledge learnt from previous tasks. The working of OAMC model is shown in Algorithm 1. The set of documents in current domain are *D* ^*t*^ = {*D*_1_, *D*_2_,…, *D*_*n*_}. *V* is the vocabulary of the current task while *N* is the number of iterations for Gibbs sampler [13] used as inference technique. Knowledge-base has the knowledge rules as must-links and cannot-links. The knowledge-base has a variety of must-links and cannot-links learnt through experience. In step (2) only those must-links and cannot-links are selected that are relevant to the current task; that is, both the words in must-link and cannot-link exist in the vocabulary of the current domain. The rules relevant to the current domain are stored as* relRules*. If no relevant rules are found, the current task is processed with baseline LDA as shown in step (4). In step (4) *∅* means that no relevant knowledge is found for the current task and therefore the baseline LDA model is to be used for the current task. It may happen at low experience when the knowledge-base has limited rules and the current task is about a completely different subject. When relevant rules are found, step (7) has Gibbs sampler [20] used for incorporating knowledge to guide the inference. The knowledge-base maintains itself after each task to be consistent. Step (9) refines* relRules* to grow their confidence and utility as a token of their services for the current domain. All rules in the knowledge-base get a task older. As part of the continuous learning process, the model harvests new knowledge rules after processing each task, in step (10). The new rules are merged with the existing rules and are added to the knowledge-base, which is ready to process a new task.

### 4.1. Knowledge Representation

**Figure 2:**
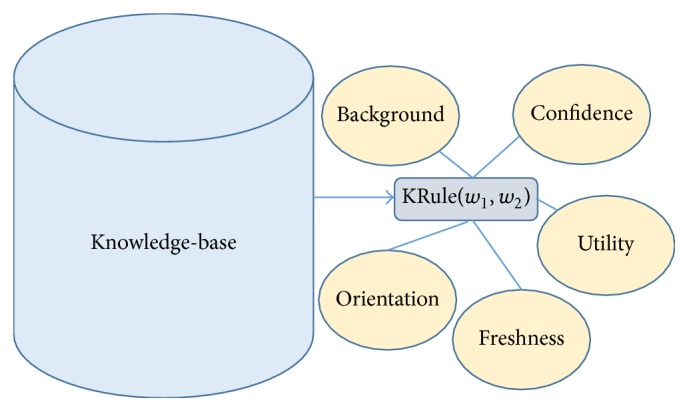
Representation of a knowledge rule as word pair in knowledge-base. Utility, freshness, and background are the newly introduced knowledge features to monitor their quality.

Additional features are added to knowledge rules to help maintain the knowledge-base and keep it consistent by continuously weeding out wrong or irrelevant knowledge. In previous models the knowledge rules are maintained as separate lists of must-links and cannot-links, each with a word pair and a confidence value. Our OAMC model stores them together as knowledge rules irrespective of their orientation. However, the orientation is communicated through a positive or negative sign used with the confidence value. New rule features are added as utility, freshness, and background to strictly monitor the contribution of each rule. To survive longer, a knowledge rule kRule(*w*_1_, *w*_2_) needs to maintain high utility in the tasks that follows. The confidence of a rule is also expected to be assured by future domains which adds to the net confidence. The utility and freshness of a rule maintain a check on its contribution and relevance. A rule with high confidence but low usage and freshness is also expected to be filtered. These newly added features keep the knowledge-base consistent with quality knowledge. The background feature helps to resolve word sense disambiguation by providing the context of the knowledge rule. A knowledge rule in the knowledge-base is stored as shown in Figure 2.

### 4.2. Knowledge Extraction

The proposed model has access to only limited big data for a task, that is, domain of the current task. Therefore, the model does not have enough instances to verify the quality of knowledge and is expected to learn low quality knowledge as well. A knowledge rule can be of low quality if it has enough support in data from where it was learnt but is not supported by future tasks. It may happen due to noise or high bias in a domain. The refinement technique deals with it until it is removed. In order to extract new knowledge rules kRule(*w*_1_, *w*_2_) normalized PMI [23] as nPMI(*w*_1_, *w*_2_) is used as given in

(1) $$\begin{array}{c} kRule\left(\;w_{1},w_{2}\;\right)=nPMI\left(\;w_{1},w_{2}\;\right)=\frac{PMI\left(w_{1},w_{2}\right)}{\log p\left(w_{1},w_{2}\right)}, \end{array}$$

where nPMI gives a value [−1,1] range. All candidate word pairs are evaluated through nPMI. For a word pair if the score is close to 1, the knowledge rule (must-link) is stored with its nPMI score as confidence. Word pair having it close to −1 is stored as a negative rule (cannot-link) with nPMI score as confidence. PMI in (1) can be evaluated as

(2) $$\begin{array}{c} PMI\left(\;w_{1},w_{2}\;\right)=\log\frac{p\left(w_{1},w_{2}\right)}{p\left(w_{1}\right)p\left(w_{2}\right)}, \end{array}$$

where *p*(*w*_1_, *w*_2_) is the coexistence probability of *w*_1_ and *w*_2_, while *p*(*w*_1_) and *p*(*w*_2_) are probabilities of their existence in the current domain. Coexistence is the presence of two words in a conceptual document irrespective of their frequency. Calculating coexistence probability and individual probabilities is shown in (3), where *D* ^*t*^ show all documents while #*D* ^*t*^ is total number of documents,

(3) pw1,w2=#Dtw1,w2#Dt,pw=#Dtw#Dt.

Freshness and utility are initialized while topic of the word pair is added to the background of the rule. The two thresholds *μ*_1_ and *μ*_2_ used are set to harvest word pairs with nPMI scores at either of the two extremes, given in

(4) $$\begin{array}{c} nPMI\left(\;w_{1},w_{2}\;\right)=\left\{\begin{array}{cc} \langle \mu_{1}\vee \rangle \mu_{2} & learn\\ else & ignore. \end{array}\right. \end{array}$$

When nPMI gives a value that is too high, that is, close to 1, or too low, that is, close to −1, then it is considered to be of significance. This untypical behavior is learnt as knowledge while the values close to zero are ignored as they have not shown any considerable corelation. Word pairs having nPMI below *μ*_1_ are learnt as knowledge (cannot-links) while the values above *μ*_2_ are also learnt (must-links). The threshold values are mentioned in Setup. Our contribution in knowledge extraction is to use nPMI(*w*_1_, *w*_2_) as a very lightweight extraction mechanism. Secondly both must-links and cannot-links are extracted together in a single step. MS-FIM was previously used to harvest knowledge which was causing a performance bottleneck. The rule mining mechanism is mentioned in Algorithm 2. It generates word pairs within each topic and evaluates them for possible knowledge from step (2) to step (11). Satisfying either of the thresholds in step (4) and step (7), the word pair is mined as positive or negative knowledge rule.

### 4.3. Knowledge Transfer

The knowledge transfer mechanism selects relevant knowledge rules each time a word is sampled to have its probability increased under a topic. The effect of relevant knowledge rules is added as bias into the topic model to push up positively corelated words under the same topic and vice versa. Since the current task has limited data, knowledge from previous tasks is incorporated to guide the inference. For a sampled word *w*_1_ in topic *t*, if a rule exists for it then the associated word *w*_2_ has probability updated in current topic *t* as shown in

(5) $$\begin{array}{c} p\left(\;{rule}_{i}=rule\;|\;t\;\right)\propto p\left(\;w_{1}\;|\;t\;\right)\times p\left(\;w_{2}\;|\;t\;\right), \end{array}$$

where *w*_1_ and *w*_2_ are the two words of a rule rule_*i*_ (must-link or cannot-link). When the model inference increases the probability of a word *w*_1_ for a topic *t*, the probability of *w*_2_ is increased for topic *t* in case of must-link and decreased for cannot-link. To address word sense disambiguation the following equation is proposed:

(6) $$\begin{array}{c} \frac{\#\left(V\cup \text{ruleBackground}\right)}{\#\text{ruleBackground}}>\xi . \end{array}$$

Here *ξ* is the threshold that has to be satisfied for overlap between the vocabulary of the current task and the rule background. The rule background represents the set of words in topic from which the rule was learnt. Comparing the rule background with the current vocabulary is to ensure that the rule is used in the same context in which it was mined. For example, the word* Bank* has different contexts when used with* Accounts* and* Sea*, respectively. Topics of all processed tasks were previously used for the same purpose. The net confidence and utility of a knowledge rule are used to transfer the impact of knowledge rule in topic sampling. Multigeneralized Polya Urn (M-GPU) model [20] is used to transfer the effect of knowledge into the current status of Markov chain in Gibbs sampler in

(7) $$\begin{array}{c} p\left(\;w\;|\;t\;\right)\propto \frac{\sum_{w^{\prime }=1}^{V}\left(\upsilon_{w,w^{\prime }}/\rho \right)\times n_{tw^{\prime }}+\beta }{\sum_{v=1}^{V}\left(\sum_{w^{\prime }=1}^{V}\left(\upsilon_{v,w^{\prime }}/\rho \right)\times n_{tw^{\prime }}+\beta \right)}. \end{array}$$

Equation (7) shows the amount of update in the probability by a factor *υ*_*w*,*w*′_/*ρ* for the other word in knowledge rule. When the probability of a sampled word is increased for a topic, using the rules, the probability of all the words associated with the sampled word is also increased. The following equation is updated to incorporate both positive and negatively corelated rules in a single step as

(8) pzi=t ∣ z−i,w,α,β,λ∝nd,t−i+α∑t=1Tnd,t−i+α×∑w′,wi∈ruleυw′,wi/ρ×nt,w′−i+β∑v=1V∑w′,v∈ruleυw′,v/ρ×nk,w′−i+β.

For word sampled *w*, the other word *w*′ sharing the same rule with it gets a push as strong as the net confidence in the direction of its orientation. The objective is to increase the probability of positively corelated words to appear together as top words under the same topic and vice versa.

### 4.4. Knowledge Maintenance and Retention

Supported with a knowledge maintenance and retention module, useful knowledge rules are stored until they justify their existence. The knowledge-base is refined after each task to keep it consistent. They all get older by a task. The relevant knowledge rules grow in confidence supported by current domain and have higher utility. After updating the state of each knowledge rule, they are passed through a threshold again to justify their existence as proposed in

(9) nPMIw1,w2=υ×ρλ>μ1  or  υ×ρλ<μ2removeotherwiseretain,

where *υ* is net confidence, *ρ* is utility, and 1/*λ* is freshness for a knowledge rule. If the confidence of a knowledge rule in current task is above its net confidence *υ*, the current domain is allowed to update the knowledge background to better represent its context, as proposed in

(10) $$\begin{array}{c} k\text{Rule}\left(\;w_{1},w_{2}\;\right)=\left\{\begin{array}{cc} conf>\upsilon & \text{update background}\\ otherwise & \text{unchanged,} \end{array}\right. \end{array}$$

where background is updated by adding words from supporting topic in the current task. New rules are mined while existing rules are evolved as a continuous process. The newly added knowledge features are used to rank and prioritize knowledge rules. The difference in rank of good and bad knowledge rules widens as the model matures with experience.  Algorithm 3 is used to refine existing knowledge, with step (2) having relevant knowledge as Rule′. In steps (4) to (7) all relevant rules get their net confidence updated and utility incremented. Step (8) has all rules aged by a task. Some of the popular rules from knowledge-base of OAMC model are shown in Table 1.

## 5. Experimental Results

The proposed model (OAMC) contributes to the application of lifelong learning for NLP tasks, specifically used to improve topic coherence for big streaming data. Various experiments are performed to highlight the improvement in accuracy and performance of lifelong topic mining as product aspects. Unlike traditional ML models it operates on big data with a variety of domains. The standard dataset used by previous LML models is used to evaluate the proposed model in comparison to the other state-of-the-art models. The data consists of 50 domains each of electronic products. To highlight the improvement in the OAMC model the dataset was provided with decreasing number of domains. The models are evaluated in topic coherence for accuracy and time in minutes for performance. The number of knowledge rules is used as quality of knowledge where fewer rules producing better results are considered to be of better quality.

### 5.1. Setup

To set up the test environment an HP Pavilion DM4 machine is used with Intel 2.4 GHz Core i5, having 8 GB RAM and 500 GB hard drive. Following the evaluation approach in existing models, 15 topics are extracted per domain for all models, while, as in previous models, top 30 words per topic are considered to calculate topic coherence of the proposed model also. The hyperparameters are kept the same for all models. The number of Gibbs sampler iterations is kept the same (2000) as used in previous models. The models are configured and initialized as used in their original work. The proposed OAMC has its thresholds *μ*_1_ and *μ*_2_ set to 0.8 and −0.9, respectively, to learn knowledge and select relevant knowledge for a given task. Since negative corelations are too many as compared to positive rules, the threshold is set to focus more on positive rules. In order to choose relevant rule the overlap threshold is set to 0.3 between the vocabulary and knowledge background.

### 5.2. Results

The accuracy of lifelong models and topic models in general is evaluated through topic coherence. It has high corelation with human evaluation. All of the models are given the same number of documents in a domain for both learning and evaluation. The existing models drop accuracy when it has to learn from a smaller dataset with documents in hundreds per domain while compromising accuracy when evaluated on larger dataset with thousands of documents. Therefore, the previous models were given large datasets to learn from and were evaluated on smaller datasets. However, for fairness to all models, they are provided with the test set only to learn from and to test on.

**Figure 3:**
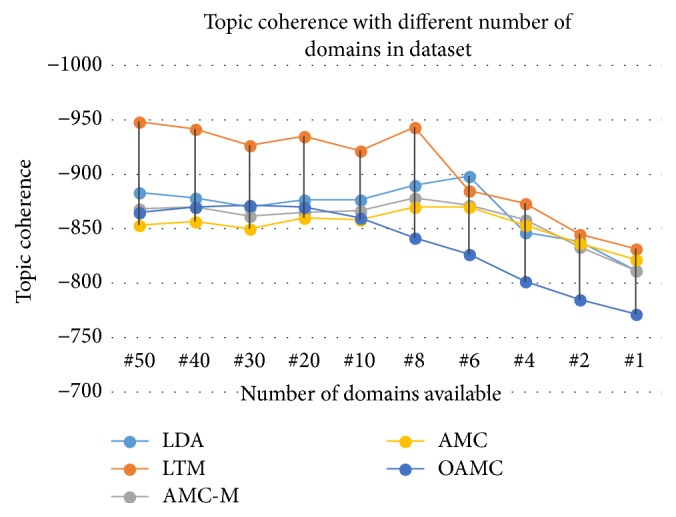
The proposed model (OAMC) produces highest accuracy (as topic coherence) among lifelong learning models for streaming big data.

Topic coherence of the models compared has interesting outcome. LTM surprisingly could not maintain a better topic coherence when it had to learn from a smaller dataset. It even dropped below the baseline. AMC and AMC-M performed well; however, the proposed OAMC beat them when a number of domains in the dataset were dropped below 10 as shown in Figure 3. AMC and AMC-M hardly learn anything with fewer domains which makes them ineffective towards streaming data. Therefore, their accuracy continuously went down towards baseline. On the other hand LTM has its learning pattern badly affected by limited variety of domains and learnt wrong knowledge which dropped its accuracy. The knowledge rules learnt by AMC and AMC-M were 100 and below per domain for less than 10 domains in the dataset which grow up to more than 2000 for 50 domains. It is because of the overreliance of the existing models on large volumes of data to be available at once, which makes them least effective towards streaming data. It shows that they only learn better knowledge and produce better results when there are many domains in the dataset and they are all available prior to analysis. With a robust knowledge storing mechanism OAMC learnt more knowledge, refined it, and used it to improve topic coherence with few domains available. The OAMC model shows on average 7% with a maximum of 10% improvement in topic coherence when there is one domain at a time.

**Figure 4:**
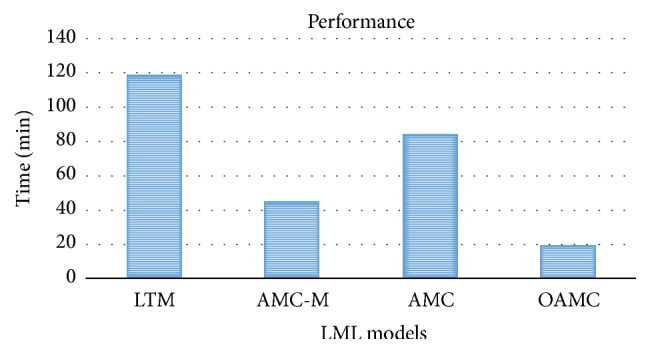
The proposed model (OAMC) has the highest performance efficiency among LML topic models.

OAMC learns wrong knowledge too as it considers only the current domain for harvesting it. However, with a robust knowledge retention module, after performing few tasks only good quality knowledge survives through the filtering mechanism. Knowledge rules with less confidence from future tasks or limited utility are pushed down and finally filtered out. Knowledge that is getting confidence reenforced from future tasks and has growing utility stays longer. To evaluate relevance of a knowledge rule with the current task, the domain of the current task must have the rule words, confidence above threshold, and overlap with the rule background higher than threshold. The online OAMC models truly learn like humans having no idea of future tasks, unlike existing models. Its learning is dependent on the past experience as the domains processed only and uses it for a future task. It is less dependent on data, parses it once, and considers the knowledge module with abstract rules only to sample relevant rules and therefore shows improvement of up to 50% in performance as shown in Figure 4.

For the discussed intrinsic evaluation, the models are also compared through PMI mean and median values that had relevance with topic coherence values. The extrinsic WordNet based techniques used in the literature (*WuPalmer, Resnik, Path, Lin, LeacockChodorow, Lesk, JiangConrath, *and* HirstStOnge*) were inconclusive to differentiate among the qualities of topics extracted due to the specialized nature of electronic products domains.

### 5.3. Evaluation

**Figure 5:**
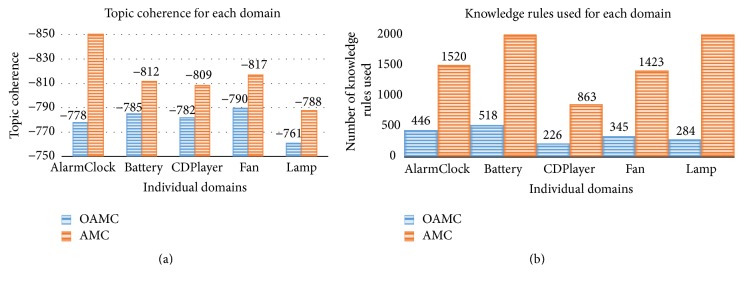
The proposed model (OAMC) in comparison to the state-of-the-art LML model (a) produces better accuracy as topic coherence for individual domains while (b) using fewer knowledge rules.

OAMC improving performance of AMC by 50% as shown in Figure 4 is attributed to the following reasons. OAMC does not extract relevant knowledge for each task and rather chooses from a well maintained knowledge module. Each domain is parsed only once. With a knowledge filtering mechanism only good quality knowledge rules are maintained. There are generally two performance bottlenecks in existing LML models, that is, FIM for knowledge extraction and higher iterations of Gibbs sampling. The OAMC model uses nPMI [23] which is highly efficient to extract knowledge. Secondly it learns both must-links and cannot-links together with a single mechanism as rules. Even with 50 domains in the dataset OAMC performed better than AMC for certain individual domains by producing higher topic coherence while using fewer knowledge rules as shown in Figure 5. Table 1 shows some of the most frequently used knowledge rules of OAMC. Through manual evaluation of topics from all the domains, it was observed that the knowledge learnt by LML models is more effective towards random and intruded types of topic incoherence but less effective towards chained and unbalanced type of incoherence. In fact the knowledge-based models can at times enhance chained topic incoherence if there are many knowledge rule pairs sharing common words.

### 5.4. Knowledge Analysis

The accuracy of LML models depends upon the quality of its knowledge. The previous models did not provide a discussion on the quality of knowledge. In fact improved topic coherence was considered as reason for good quality knowledge. The knowledge rules shown in Table 1 have utility above 10 as a token of their quality. The ratio of use of knowledge rules per domain by AMC and OAMC is 5 : 1, signifying the quality of knowledge produced by OAMC. Net confidence of rules also shows that most of the relevant domains enforced the rule and increased its confidence (above 0.8 with 1 as maximum). Since this information is not available for the existing models, the values cannot be matched. It is still very difficult to explore the impact of each knowledge rule separately; however, the discussed features in general keep track of good quality knowledge. The knowledge learnt is of two types only capturing positive and negative word corelations. However, more varieties of knowledge can be introduced for further insights. By introducing “the type of relationship” as knowledge, the unbalanced topic incoherence problem can be addressed. Along with correlation between words, the nature of their correlation may also be explored for better analysis. This type of knowledge is also useful for finding hierarchical topics. The knowledge rules extracted are isolated, while knowledge as complex network can be more effective for a variety of analyses. AMC uses a small graph only to associate must-links sharing common word in the same context. Knowledge extraction, transfer, representation, and retention are only introduced to NLP tasks in 2010 and require mature ideas from other fields of research.

## 6. Conclusion

LML models are structured to process big data having many domains and therefore it is the only suitable solution, despite its shortcomings. LML models are recently introduced to big data topic extraction and are far from mature. The existing LML topic models had limited application due to following batch processing approach and overreliance on data. Our proposed model named as OAMC follows online approach for LML topic modeling that can support streaming data. With an efficient learning mechanism our OAMC model has lower dependency on data and therefore improved performance by 50% as compared to state-of-the-art LML models. Velocity is an important feature of big data as it is believed to be received continuously at high speed. The OAMC model is designed to process the data in hand only and therefore outperforms existing models for streaming data. As the data is expected to have noise, OAMC is supported with a strict monitoring and filtering mechanism to maintain a consistent knowledge-base. The rules are made to compete for survival based on utility, freshness, and net confidence. OAMC being blind to the future gets higher bumps in accuracy when current task has low relevance to past experience. This is very common to a human-like learning approach. However, the tendency of such tasks for which limited relevant knowledge available drops with experience considerably. OAMC model shows 7% improvement in accuracy on average for streaming big data but up to 10% for certain individual domains as compared to state of the art.

In the future we will be working on introducing more varieties of knowledge. Through complex network analysis the knowledge rules can be stored in association with each other. Cooccurrence can mean many things and, therefore, the relationship type should also be considered in future. It will help to associate aspects with products, sentiments to aspects, and reasons to sentiments. To improve the performance and attempt for near real-time analysis, Gibbs sampling should be replaced with a lighter inference technique.

## Figures and Tables

**Algorithm 1:**
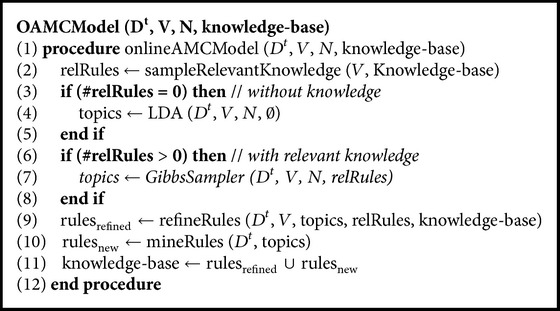
OAMC model.

**Algorithm 2:**
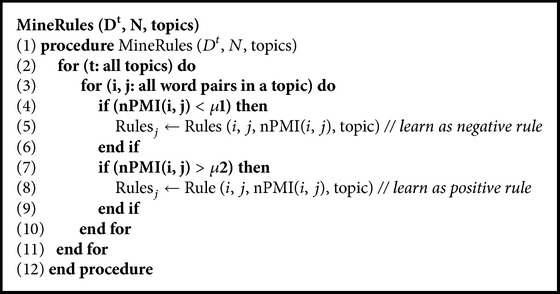
Mine rules.

**Algorithm 3:**
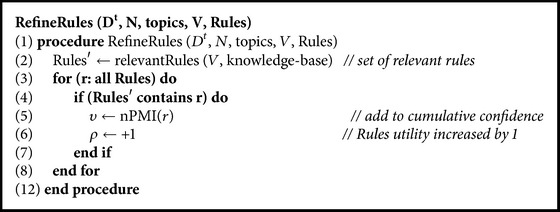
Refine rules.

**Table 1:** OAMC positive and negative knowledge rules with high utility.

Type	Knowledge
+ive	(tech, support), (cable, usb), (pro, con), (customer, support), (batter, charge), (video, card), (high, long), (connection, vga), (operating, system), (monitor, macbook), (mac, support), (cd, dvd), (windows, xp), (money, worth), (inch, picture), (input, output), (friend, mine), (big, deal)

−ive	(sound, money), (feature, battery), (battery, price), (feature, battery), (price, device), (screen, sound), (review, screen), (worse, digital), (price, easy), (gas, screen), (signal, easy), (home, gps), (hotel, traffic), (driver, cooler), (monitor, processor), (monitor, speed)
